# Relative Permittivity Measurement of Microliter Volume Liquid Samples through Microwave Filters

**DOI:** 10.3390/s23062884

**Published:** 2023-03-07

**Authors:** Azhar Yasin, Nayab Gogosh, Syed Irfan Sohail, Syed Muzahir Abbas, Muhammad Farhan Shafique, Abdelhady Mahmoud

**Affiliations:** 1 Department of Electrical and Computer Engineering, COMSATS University Islamabad, Islamabad 45550, Pakistan; 2 Department of Computing and Technology, IQRA University Islamabad Campus, Islamabad 44000, Pakistan; 3 Faculty of Science and Engineering, School of Engineering, Macquarie University, Sydney, NSW 2109, Australia; 4 Center for Advanced Studies in Telecommunication, COMSATS University Islamabad, Islamabad 45550, Pakistan; 5 Benha Faculty of Engineering, Benha University, Benha 13512, Egypt

**Keywords:** dielectric liquids, dielectric measurements, microwave filters, substrate-integrated waveguides (SIW)

## Abstract

This paper proposes a concept of dielectric characterization of low-volume liquid samples using the coupling coefficient of filters. The concept is validated through a two-pole substrate integrated waveguide filter in which the liquid under test is mounted on the coupling section between the two resonators. Unlike the conventional resonator perturbation method reported many times in the literature, this technique uses the coupling coefficient for sensing. The liquid sample is collected in a capillary tube and carefully positioned on the coupling section of the filter; the coupling coefficient of the two resonators varies compared to the relative permittivity of the sample; thus, an empirical model is established. The proposed sensor has been tested to compute the permittivity of different alcohols. Binary solutions of ethanol and water have also been characterized to calculate the volume ratio and relative permittivity as a proof-of-concept. The obtained results show that the proposed sensing technique is capable of characterizing a low quantity of liquids (≈44 µL) with good accuracy, and a worst case measured error of only 6.8% is noted. The ease of integration with other circuitry, low cost, reusability with no deterioration, and adaptability of the proposed sensor makes it a suitable choice for the chemical as well as for the pharmaceutical industry.

## 1. Introduction

With the theoretical advancements in electromagnetics, several investigations have been carried out to assess the behavior of materials under the influence of electromagnetic fields. The electromagnetic characterization of materials is as old as the theory of electromagnetics itself. One of the earlier works on the dielectric characterization of materials was reported way back in 1895 [1]. Since then several techniques based on resonant as well as non-resonant structures have been developed to estimate the dielectric properties of materials [2]. Resonant sensors offer a higher level of accuracy at a discrete set of frequencies, whereas non-resonant structures can be used to characterize materials at a wider range of frequencies, but with low accuracy [3].

The resonator perturbation method is based on the perturbation approach, in which the material under test is loaded to the resonator in such a way that the resonator deviates from its real resonant frequency. This change in the resonant frequency is a function of the permittivity of the material under test (MUT) [4]. Numerous structures for characterizing solids with planar and nonplanar sensors have been proposed [5,6,7,8]. Recent work on dielectric measurements of dielectric sheets has been reported in [9]. The sensing element is based on complementary split-ring resonator (CSRR) cells etched into the ground plane of a microstrip line. This sensor has been used to test various dielectric sheets and a worst case error of 6.92% has been reported.

Liquid characterization, on the other hand, is challenging since it requires a contained environment. In the chemical and pharmaceutical industries, liquid characterization is essential, and there remains a significant demand for highly accurate sensors capable of characterizing liquids [10,11,12,13]. In this regard, researchers in [14] describe a sensor based on a cylindrical cavity with a diameter of 76.66 mm and a height of 40 mm operating in TM_010_ resonant mode at a frequency of 3 GHz for the dielectric characterization of liquids such as water, methanol, and ethanol. A 2 mm capillary tube was used to hold the sample and was inserted into the cavity. The permittivity was calculated using field analysis and perturbation methods, and a worst case error of 8% was obtained for a methanol sample using the cavity perturbation approach. Likewise, [15] describes another type of sensor that uses dielectric resonators (DR) as its primary sensing element. The reported sensor operates at a resonant frequency of 10.5 GHz in TM_01δ_ mode. The dielectric properties of isopropanol and ethanol were characterized, and it was established that the sensor responded well to materials with low to moderate dielectric loss. However, the DR’s non-planar shape was a major limitation in this design, and the accurate placement of samples for repeated measurement was another issue. The worst case error was recorded at 7.84%. A better approach for liquid characterization using a split-ring resonator (SRR) was presented in [16]. The SRR was fabricated by bending a silver-coated copper wire and the liquid under test (LUT) was placed in a capillary tube between the coupling section of the SRR. The device offered decent sensitivity and the worst case reported error was 5.12%, but the sensor was not easily integrable with other electronics.

With technological progress and miniaturization in the electronics sector, the requirements have shifted to the development of compact and planar sensors. For this purpose, a number of structures have been reported that are not only compact but allow straightforward integration with other assistive electronics. Recent work in this regard proposes a sensor based on a quartet of CSRRs arranged in a honeycomb configuration beneath a microstrip line. This sensor was employed to detect the glucose content in the human body, and water–glucose solutions were used in these experiments to mimic blood behavior at different glucose concentrations. A glass jar containing a precise amount of LUT was loaded onto the CSRR cell for each measurement. The technique was efficient but it required a large amount of LUT [17]. Likewise, another study was carried out that was based on a CSRR element. The CSRR was etched in the ground plane of the microstrip line and the capillary tube containing the LUT was introduced longitudinally through the substrate. The LUT placement was very challenging in this setup and a worst case error of 8.38% was reported [18]. Similarly, the technique of interdigitated capacitance has been used with SRR to enhance the sensing area [19]. Small quantities of liquid was pushed into a polydimethylsiloxane (PDMS) microfluidic channel through a syringe. Ethanol–water and methanol–water solutions were characterized and the worst case error of 8.03% was observed. Etched CSRR in a microstrip line’s ground plane was suggested as an alternative sensing method [20]. A PDMS holder was mounted on the sensor and the LUT was pushed through it. A maximum error of 10% was observed in the measurements of a water–ethanol solution. Characterizing liquids is also the focus of another study that makes use of a double SRR construction. The sensor featured a plastic pipe segment mounted on its outer surface to contain the LUT. A worst case error of 8.7% was determined in the testing of a water–methanol mixture [21].

Exploring further the miniaturization possibilities, another recent study discussed a planar sensor based on an impedance network [22]. An open CSRR serves as the basis for this design. Using the proposed sensor, the characteristics of deionized water and ethanol were determined, and the largest error observed was 11.25%. Investigating further the domain of planar sensors, a microstrip square ring resonator that can sense liquids in trace amounts at discrete frequencies was suggested in [23]. The field perturbation method was adopted for various liquid samples at 1 GHz, 2 GHz, and 3 GHz. The worst case error for 1-propanol measurements at 1 GHz was recorded at 13.18%. A 2.4 GHz microstrip resonant sensor was developed using a ground plane slot [24]. This slot was built into the base of the microstrip resonator and had a width of 1 mm. To accurately characterize liquids, the slot was required to be completely submerged in a binary liquid solution, such as ethanol and methanol. The largest error recorded by the authors was 4.4%.

Furthermore, researchers have investigated the potential application of substrate-integrated waveguide (SIW) technology for dielectric characterization. Owing to the high quality factor of SIW, it has the potential to be used as a sensing element. It has been established that the SIW cavity sensor with an 8 GHz resonance can successfully analyze liquids [25]. As a sensor, the SIW cavity had dimensions of 23 mm in length, 15 mm in width, and 2.8 mm in thickness, which was smaller than a hollow waveguide resonating at the same frequency. The sensor provided great precision with a deviation of ±0.5%; nevertheless, the sample had to be mounted inside the cavity using a capillary tube, and a snug fit was required to prevent air gaps. A SIW cavity that operates in TE_102_ mode was produced in another work [26]. An aperture was constructed on the top metal surface of the cavity to allow radiations to leak out of the cavity, and the resulting sensor was submerged in a jar that was filled with the LUT. The maximum error calculated was 5%. A sixteenth-mode SIW cavity resonator for liquid sample characterization is reported in [27]. Similar to other sensors its sensing mechanism was also based on the resonant perturbation method. A hole was drilled at the maximum E field location and the LUT was filled in the hole to measure the dielectric properties.

All of the methods reported above are based on the premise of the resonator perturbation theory, in which the introduction of the LUT disrupts the internal fields of the resonator and varies its natural resonant frequency.

In this study, we suggest a different approach for characterizing liquids. In lieu of perturbing the resonator field distribution, the coupling coefficient between the resonators of a filter has been disrupted by mounting the LUT. For this purpose, an SIW filter was utilized. When LUT is placed on the filter’s coupling section, it alters the coupling coefficient (K) between the resonant elements. On the basis of the filter sensitivity and permittivity of the LUT, the coupling coefficient changes, thus allowing for the determination of the permittivity/composition of the LUT. The proposed method can be easily applied to additional filter topologies, such as microstrip and waveguide filters. The devised technique does not vary the operating mode of the SIW cavity resonators, but rather changes the coupling between them. The conventional resonators have sensitivity limitations that are directly related to their unloaded quality factors. On the other hand, this technique relies on coupling sensitivity rather than the quality factor of the resonator, which opens up new opportunities for investigating different filter configurations.

## 2. Geometry of SIW Filter

**Figure 1:**
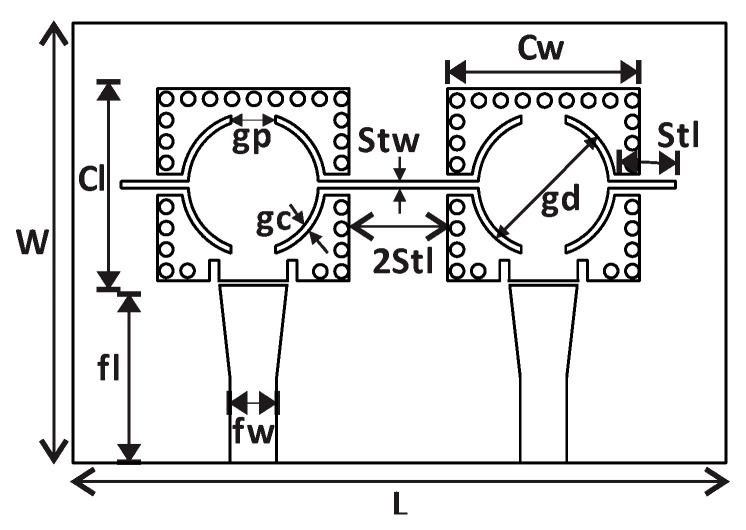
SIW filter with loosely coupled feed lines; dimensions are (in mm): L = 47, W = 32, Cl = Cw = 14, fl = 12.75, fw = 3.63, gp = 3.6, gc = 0.5, stw = 0.5, stl = 4.4, gd = 10.5.

To validate the proposed concept of perturbing the coupling coefficient between two resonators, a compact two pole SIW filter was adopted [28]. The basic element involved in the design of this filter (Figure 1) was a resonant cavity that was etched with C shape slots and loaded with two inductive stubs on the side walls.

The two resonators in the filter were coupled through inductive stubs which were connected to the side walls. In this side-by-side configuration, the connected stub in the middle controlled the coupling coefficient between the two resonators, and by modifying the length or width of the connected line, the coupling coefficient was varied. The coupling coefficient was calculated with the relation given in (1) [29,30].

(1) $$K_{ij}=\frac{f_{h}^{2}-f_{l}^{2}}{f_{h}^{2}+f_{l}^{2}}$$

where:

*f_h_* and *f_l_* are the peaks of the transmission coefficient when the source and load of the filter are loosely coupled.

**Figure 2:**
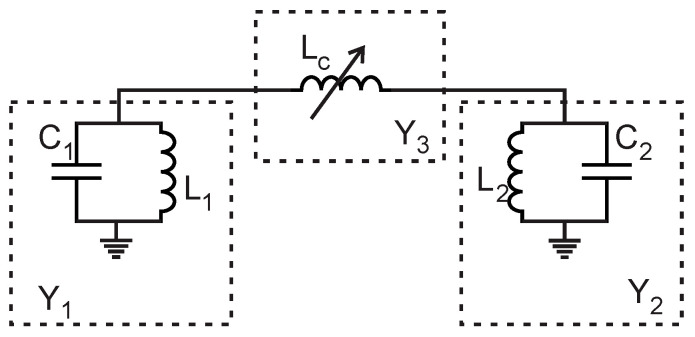
Simplified equivalent circuit model.

It can be observed from Figure 1 that the feeding lines were not connected to the filter directly and a capacitive gap was introduced. This technique supported the clear identification of two resonant peaks (even and odd modes) in the transmission coefficient of the measured results. The simplified equivalent circuit model of the filter is presented in Figure 2. The coupling section is represented by the inductor only for simplicity; this inductor (Lc) varies when the coupling section is loaded with the LUT.

The network can be simplified using the admittance parameters shown in Equations (2)–(4). The resulting ABCD matrix can be written as (5) [30] and further simplification of the matrix is presented in (6) and (7).

(2) Y1=jωC1+1jωL1

(3) Y2=jωC2+1jωL2

(4) Y3=1jωLc

(5) [ABCD]=[1+Y2Y31Y3Y1+Y2+Y1Y2Y31+Y1Y3]

(6) $$\left[\begin{array}{cc} A & B\\ C & D \end{array}\right]=\left[\begin{array}{cc} 1+\frac{L_{c}}{L_{2}}\left(1-\omega^{2}C_{2}L_{2}\right) & j\omega L_{c}\\ U & 1+\frac{L_{c}}{L_{1}}\left(1-\omega^{2}C_{1}L_{1}\right) \end{array}\right]$$

where

(7) $$U=\frac{1}{\omega L_{1}L_{2}}\left[L_{c}\left(\omega^{2}C_{2}L_{2}\left(\omega^{2}C_{1}L_{1}-1\right)+1\right)-\omega^{2}C_{1}C_{2}L_{1}+j\left(L_{2}-L_{1}+\omega^{2}L_{1}L_{2}\left(C_{1}+C_{2}\right)\right)\right]$$

**Figure 3:**
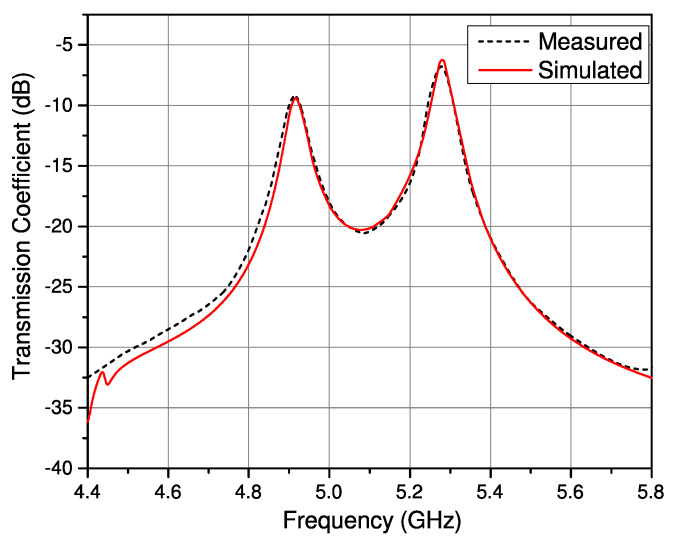
Simulated and measured transmission coefficient of the SIW filter.

The simulated and measured results of the filter are shown in Figure 3.

The transmission coefficient response of the filter shows two distinct peaks representing the resonant frequency response of the even and odd modes of the two resonators. This response was attained by keeping the coupling gap between the transmission lines and the resonators. If the feeding line is connected, a flat transmission response would not allow the accurate identification of the peaks which are necessary for further characterization.

## 3. Liquid Characterization Procedure

### 3.1. Sensitivity Analysis

**Figure 4:**
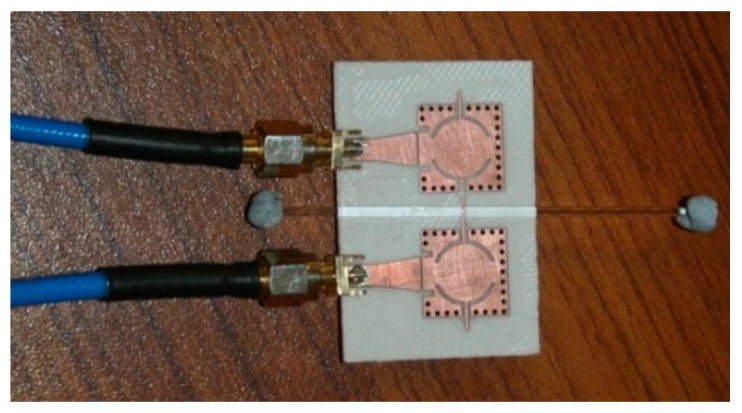
The vertical placement of the LUT on the SIW filter.

**Figure 5:**
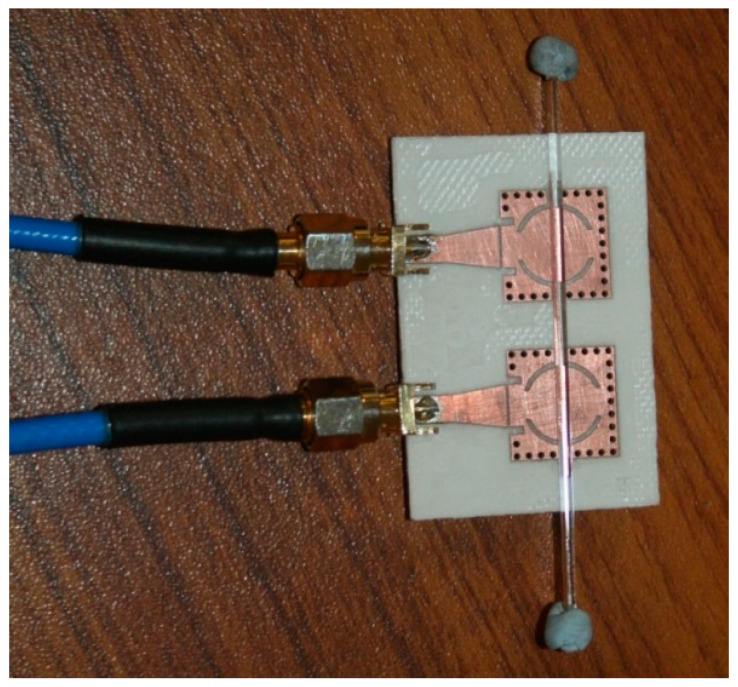
Horizontal placement of the LUT on the SIW filter.

As discussed earlier, the connecting stub is responsible for controlling the coupling coefficient of the filter. It has been found that loading this coupling stub with the LUT will affect the effective permittivity of this section, which in turn will cause a change in the coupling coefficient. The coupling section consisted of a connected stub between the resonators and the fringing fields of the stub interacted with the sample when it was placed on it. Glass capillaries filled with different analytical research grade chemicals were mounted on this coupling section in vertical and horizontal orientations, as illustrated in Figure 4 and Figure 5 to analyze the sensitivity of the structure. The transmission response of the filter was then measured using a vector network analyzer (Agilent N5242A). The glass capillaries used in this work had an inner diameter of 0.8–1.1 mm and an outer diameter of 1.5 mm. The actual length in contact that causes the measurable shift was 47 mm (the width of the substrate). Thus, the minimum quantity of liquid required in the capillary was 44 µL. The tube was made of borosilicate glass with a relative permittivity of 3.4 and a loss tangent of 0.0015 [25].

**Figure 6:**
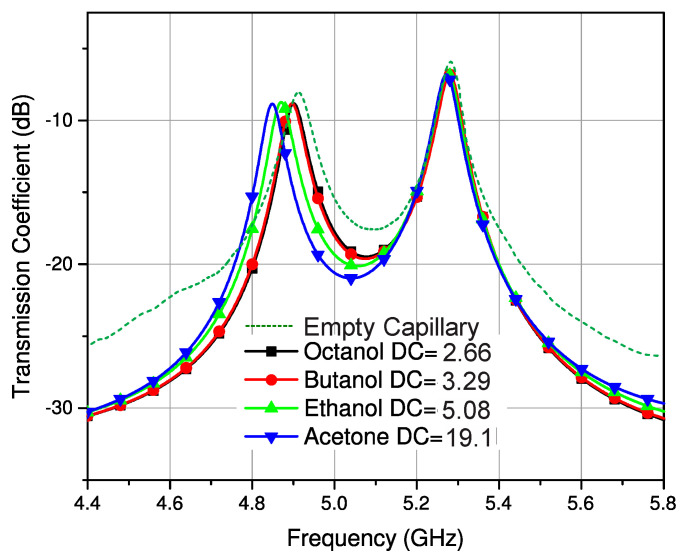
Response of filter with the vertical orientation of the LUT.

**Figure 7:**
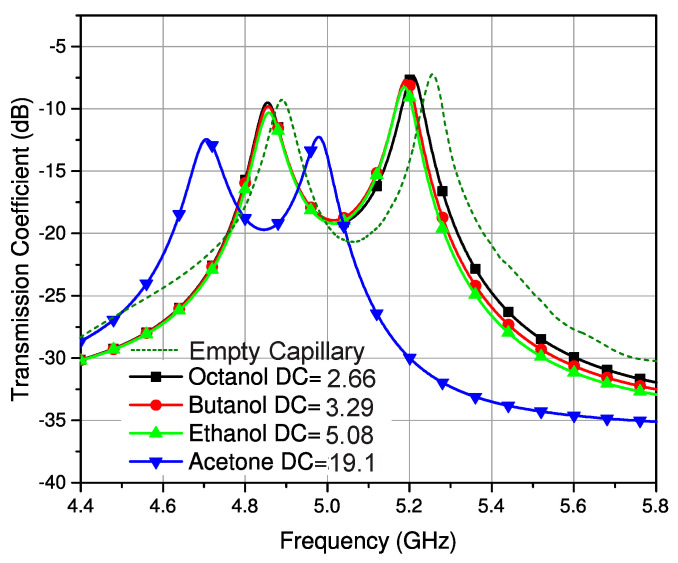
Response of filter with the horizontal orientation of the LUT.

It can be observed from the measured results shown in Figure 6 and Figure 7 that the samples of different liquids placed horizontally on the SIW filter offer a more distinguishable response and better sensitivity compared to the samples placed on the filter vertically. The larger section of the coupling stub interacts with the sample in a horizontal configuration, thus offering more distinct variation. It is also evident that the coupling coefficient undergoes a larger change for high permittivity liquids. The standard values of the liquids were taken from different sources at 20 °C [31,32,33]. The room temperature was regulated to 20 °C for all these experimentations and an average of five readings was considered for all measurements. The next stage was to determine the composition of a mixture with different concentrations of binary liquids. In this regard, a known mixture of ethanol and distilled water was used.

### 3.2. Sample Preparation

In order to prepare reference samples, analytical research grade chemicals (MERCK chemicals) were used and various solutions of ethanol–distilled water with different known concentrations at 20 °C were prepared. The dielectric properties of the prepared solution in different concentrations were theoretically calculated using the dielectric mixture relation as given in (8) [34,35,36].

(8) $$\varepsilon_{r}\left(f\right)=\varepsilon_{1\;}\left(f\right)\left[\frac{\left\{2\varepsilon_{1\;}\left(f\right)+\varepsilon_{2}\left(f\right)\right\}+2q\left\{\varepsilon_{2\;}\left(f\right)-\varepsilon_{1\;}\left(f\right)\right\}}{\left\{2\varepsilon_{1\;}\left(f\right)+\varepsilon_{2}\left(f\right)\right\}-q\left\{\varepsilon_{2\;}\left(f\right)-\varepsilon_{1\;}\left(f\right)\right\}}\right]$$

where *ε_r_*(*f*) is the complex permittivity of solution at a specific frequency; *ε*_1_(*f*) is the complex permittivity of the entire matrix, which in this case is ethanol; *ε*_2_(*f*) is the complex permittivity of the inclusion, which in this case is distilled water; and q is the volume fraction of the solution. Values of q range from 0 to 1. For pure ethanol (entire matrix) with no water (inclusion), the value of q is 0 and it changes with the varying amounts of water being added to the matrix to a maximum value of 1, which means only inclusion. The complex permittivity values of ethanol and water were taken from the literature at 20 °C. Equation (8) is a classic mixing relation that calculates the complex permittivity of the solution based on the complex permittivities of the solute, solvent, and volume fraction. Temperature variation is known to produce permittivity changes; therefore, the effective permittivity of the solution obtained from Equation (8) would only be valid at the specified temperature. There is no direct temperature compensation mechanism available in (8) because the permittivity variation of all solvents is nonuniform with reference to the change in temperature.

### 3.3. Sensor Calibration

At 5 GHz and 20 °C, the complex permittivity of ethanol is 5.08–3.62 J, and for water, it is 73.3–21.9 J. The values of the complex permittivity of pure liquids at the frequency of operation were taken from different sources [31,37,38].

**Figure 8:**
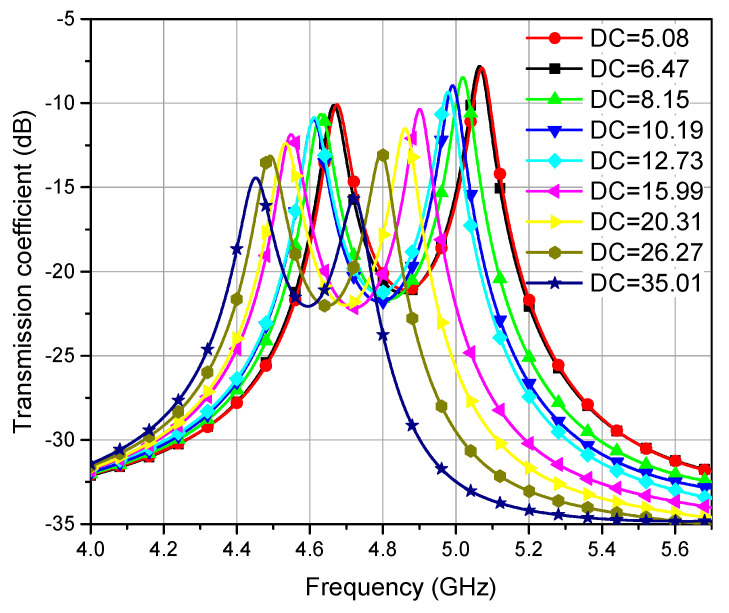
Measured response of ethanol–distilled water solution, with frequency vs. S21 for different concentrations of solutions.

**Figure 9:**
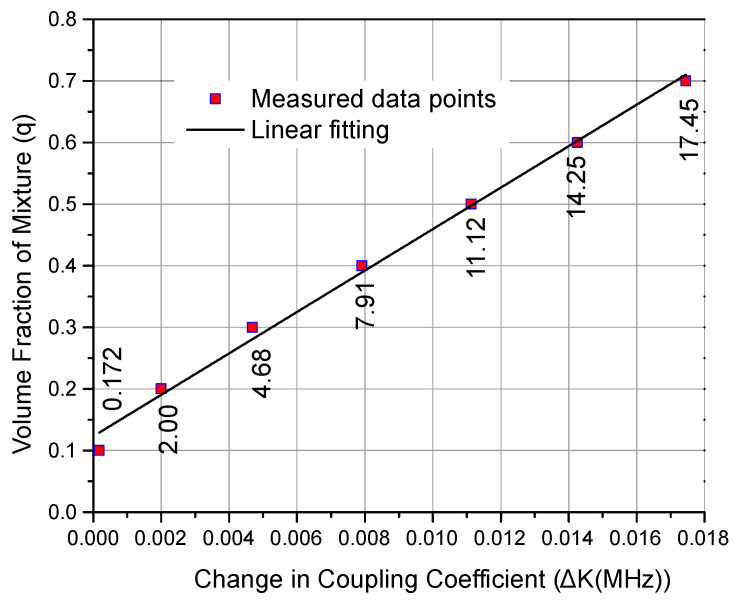
Change in coupling coefficient vs. volume fraction of mixture.

Once the calibration samples were prepared, they were mounted on the sensor and their response was measured through VNA. It was observed that each sample induced a change in the coupling coefficient of the filter to a certain extent that was observed from the transmission response of the filter. This change in the coupling coefficient (ΔK) is caused by the change in the volume fraction and, ultimately, the change in the effective relative permittivity of the solution (LUT). Therefore, the change in the coupling coefficient of the SIW filter was measured and calibrated against known volume fractions of sample solutions and their effective permittivities. The effect of the capillary tube was incorporated into the calibration process, the reference point (0 shift) was considered as the response of an empty capillary tube, and all other shifts were measured relative to this frequency point. Thus, the effect of the capillary tube was constant for all measurements. The measured transmission coefficients (S21) for ethanol–water solutions are shown in Figure 8. From this measured transmission response, Figure 9 is plotted, which relates the volume fraction (q) of a liquid with the change in the coupling coefficient (ΔK) and its mathematical relation is given in (9). It is observed from Figure 8 that the resonant frequencies of the two resonators decrease owing to the larger water content for higher volume fractions; thus, the larger effective permittivity of the mixture solution is observed.

(9) q=33.64ΔK+0.123

**Figure 10:**
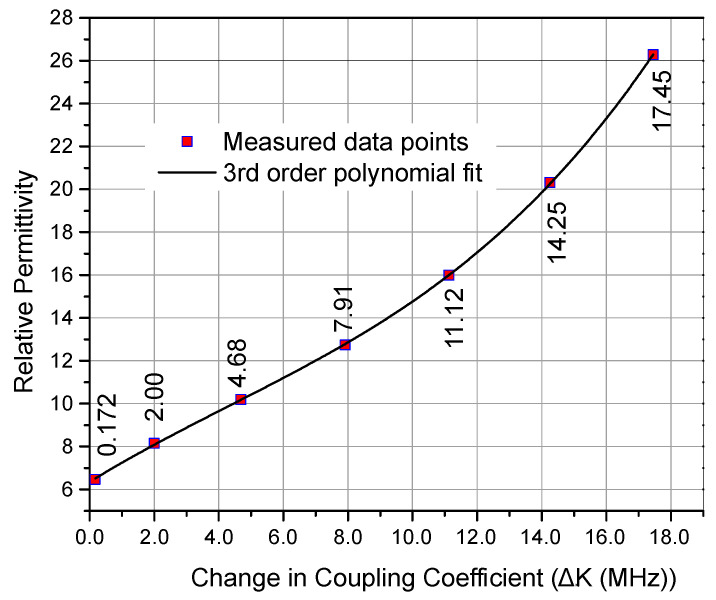
Change in coupling coefficient vs. relative permittivity of the mixture.

The above relation directly determines the unknown volume fraction of the ethanol–water solution once the change in coupling coefficient is measured for a random LUT sample of an ethanol–water solution. Next, since the relation between the change in coupling coefficient and the volume fraction was already established through the measurement of the known solution, the next step was to establish the relation between the change in the coupling coefficient and relative permittivity. The measured change in coupling coefficient was plotted against the relative permittivities of the binary mixture solutions, as shown in Figure 10. The data points were interpolated and a third-order polynomial relation has been proposed in (10).

(10) $$\varepsilon_{r}=2.8\times 10^{6}\Delta K^{3}-3.65\times 10^{4}\Delta K^{2}+926.6\Delta K+6.35$$

Equation (10) directly relates the measured change in coupling coefficient to the effective relative permittivity of the solution. Larger changes reflect the larger water content in the sample, the response is linear for lower concentrations up to 40% (q < 0.4), and then the relative permittivity increases non-linearly for higher values of volume fraction. This fact can also be observed in Equation (8) where the permittivities of both matrix and inclusion remain constant but 2q in the numerator makes the relation non-linear for larger values of q.

### 3.4. Characterizing Unknown Samples

For the measurement of unknown samples of an ethanol–water solution, the LUT was mounted on the sensor and the transmission coefficient was measured from VNA. From the measured transmission coefficient, a change in the coupling coefficient ΔK was calculated relative to the empty capillary tube reference, this change in the coupling coefficient allowed the determination of volume fraction (q) through Equation (9) and also the relative permittivity of the unknown solution through Equation (10).

**Figure 11:**
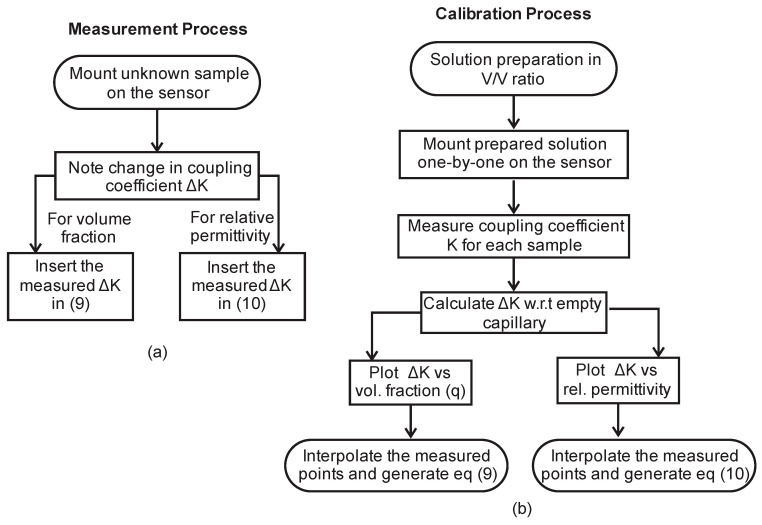
Functionality flow chart. (**a**) Steps for measuring unknown sample from pre-calibrated solution category. (**b**) Calibration steps to add a new solution category.

The measurement process and calibration process are summarized in the flow chart in Figure 11.

## 4. Measured Results and Comparison

The samples used for calibrating the sensor were used again and a number of fresh samples of the ethanol–water solution was prepared with a known volume fraction to measure their composition and permittivities through the priorly calibrated sensor. In Table 1, the measured results obtained through the proposed sensor are compared with the theoretical results computed with the mixing model. It is observed that the measured results are in excellent agreement with the mixing model values of the relative permittivity. The worst case measured relative permittivity error of 6.8% was observed, which is primarily due to cumulative errors from empirical relations. The errors are much smaller in the medium permittivity range.

The proposed technique of liquid characterization using a coupling coefficient was with other relevant techniques from the literature in Table 2. It can be seen from Table 2 that non-planar sensors are not suitable candidates for lab-on-chip or single board solutions. The planar sensing structures are either less accurate or a special mounting process is required for them. As in the case of [21], the SIW based sensor is very accurate, but it has challenging fabrication requirements, and a lateral through hole is required which holds the capillary in the middle of the cavity for maximum perturbation. This technique that we propose, however, requires no special mounting; the sample is easily placed on the surface of the substrate above the coupling section. The sample can be changed easily without any hassle.

## 5. Conclusions

A method for the efficient and rapid dielectric characterization of liquid mixtures is proposed in this work. The characterization is based on the phenomenon of coupling coefficient perturbation instead of the resonant cavities themselves. For the proof-of-concept, solutions of ethanol–distilled water with variable concentrations were prepared. The dielectric properties of the prepared solution in different concentrations were calculated through the mixing model and compared with the measured results. A worst case measured relative permittivity error of 6.8% was observed for the low permittivity range, and for medium permittivity ranges, the error was reduced to less than 3%, which shows good agreement between the measured results and the mixing model values. The proposed sensor is suitable for the pharmaceutical industry owing to the quick and straightforward sample mounting technique. The working mechanism allows the reusability of sensors many times without aging or deterioration.

## Figures and Tables

**Table 1:** Comparison of measured results with theoretical data for ethanol–water solution and error%.

Volume Fraction (q)	Relative Permittivity (ε_r′_)		
	Mixture Model [35]	Proposed Sensor	Error (%)
0.1	6.47	6.91	6.8
0.2	8.15	8.00	1.84
0.3	10.19	9.92	2.65
0.4	12.73	12.41	2.51
0.5	15.99	15.72	1.68
0.6	20.31	20.12	0.93
0.7	26.27	25.87	1.52

**Table 2:** Comparison of this work with other techniques reported in the literature.

	Sensor Type	Profile	LUT Mounting	Frequency (GHz)	Worst Case Error (%)	Merits/Demerits
[14]	Cylindrical cavity resonator	Non-Planar	Inside cavity	3	8	Large form factor and not integrable to a single board solution
[15]	Dielectric resonator	Non-Planar	Atop dielectric resonator with capillary tube	10.5	7.84	Large form factor and not integrable to a single board solution
[16]	Wired Split-Ring Resonator	Non planar	Between SRR coupling section with capillary tube	3	5.12	Large cavity-based structure and not integrable to a single board solution
[18]	CSRR backed microstrip	Planar	Inside substrate hole with capillary	2.4	8.38	Planar design but hard to manufacture, sensor deterioration
[19]	Interdigitated SRR	Planar	Inside a PDMS microfluidic channel	1.49	8.03	Planar, extra PDMS microfluidic channel is required for LUT testing
[20]	CSRR backed microstrip	Planar	Inside a PDMS microfluidic channel	2.2	10	Planar, extra PDMS microfluidic channel is required for LUT testing
[21]	Double SRR	Planar	Held in a plastic pipe segment	1.9	8.7	LUT holder is non disposable and requires thorough cleaning before every measurement
[22]	Microstrip open split-ring resonator	Planar	In a container atop the sensor	0.7	11.25	Uses impedance network, large error, a large sample size required
[23]	Microstrip square-ring resonator	Planar	On resonator with capillary tube	1, 2, 3	13.18	Compact design but has a large error and is less accurate
[24]	Microstrip resonator with a slot in the ground	Planar	Immersion in liquid	2.2–2.6	4.4	Not integrable to a single board solution, needs to be submerged in the LUT, a large sample size required
[25]	SIW cavity resonator	Planar	Inside cavity	8	±0.5	Highly accurate but difficult sample mounting.
This work	SIW filter	Planar	On resonator with capillary tube	4.8–5.3	6.8	Good accuracy, sample mounting, extremely repeatable in same environmental conditions and integrable to a single board, no deterioration, microliter sample volume is sufficient
